# Comparing relationships between health-related behaviour clustering and episodic memory trajectories in the United States of America and England: a longitudinal study

**DOI:** 10.1186/s12889-022-13785-7

**Published:** 2022-07-16

**Authors:** Jing Liao, Shaun Scholes, Claire Mawditt, Shannon T. Mejía, Wentian Lu

**Affiliations:** 1 Department of Medical Statistics, School of Public Health, Sun Yat-Sen University, Guangzhou, China; 2 Sun Yat-sen Global Health Institute, Institute of State Governance, Sun Yat-sen University, Guangzhou, China; 3 Research Department of Epidemiology and Public Health, University College London, London, UK; 4 Graduate School of Public Health, St Luke’s International University, Tokyo, Japan; 5 Department of Kinesiology & Community Health, College of Applied Health Sciences, University of Illinois at Urbana-Champaign, Champaign, USA

**Keywords:** Health-related behaviour clustering, Cognitive functioning, Cross-country comparison

## Abstract

**Background:**

Health-related behaviours (HRBs) cluster within individuals. Evidence for the association between HRB clustering and cognitive functioning is limited. We aimed to examine and compare the associations between three HRB clusters: “multi-HRB cluster”, “inactive cluster” and “(ex-)smoking cluster” (identified in previous work based on HRBs including smoking, alcohol consumption, physical activity and social activity) and episodic memory trajectories among men and women, separately, in the United States of America (USA) and England.

**Methods:**

Data were from the waves 10–14 (2010–2018) of the Health and Retirement Study in the USA and the waves 5–9 (2010–2018) of the English Longitudinal Study of Ageing in England. We included 17,750 US and 8,491 English participants aged 50 years and over. The gender-specific HRB clustering was identified at the baseline wave in 2010, including the multi-HRB (multiple positive behaviours), inactive and ex-smoking clusters in both US and English women, the multi-HRB, inactive and smoking clusters in US men, and only the multi-HRB and inactive clusters in English men. Episodic memory was measured by a sum score of immediate and delayed word recall tests across waves. For within country associations, a quadratic growth curve model (age-cohort model, allowing for random intercepts and slopes) was applied to assess the gender-stratified associations between HRB clustering and episodic memory trajectories, considering a range of confounding factors. For between country comparisons, we combined country-specific data into one pooled dataset and generated a country variable (0 = USA and 1 = England), which allowed us to quantify between-country inequalities in the trajectories of episodic memory over age across the HRB clusters. This hypothesis was formally tested by examining a quadratic growth curve model with the inclusion of a three-way interaction term (age × HRB clustering × country).

**Results:**

We found that within countries, US and English participants within the multi-HRB cluster had higher scores of episodic memory than their counterparts within the inactive and (ex-)smoking clusters. Between countries, among both men and women within each HRB cluster, faster declines in episodic memory were observed in England than in the USA (e.g., b _England versus the USA for men: multi-HRB cluster_ = -0.05, 95%CI: -0.06, -0.03, b _England versus the USA for women: ex-smoking cluster_ = -0.06, 95%CI: -0.07, -0.04). Additionally, the range of mean memory scores was larger in England than in the USA when comparing means between two cluster groups, including the range of means between inactive and multi-HRB cluster for men (b _England versus the USA_ = -0.56, 95%CI: -0.85, -0.27), and between ex-smoking and multi-HRB cluster for women (b _England versus the USA_ = -1.73, 95%CI: -1.97, -1.49).

**Conclusions:**

HRB clustering was associated with trajectories of episodic memory in both the USA and England. The effect of HRB clustering on episodic memory seemed larger in England than in the USA. Our study highlighted the importance of being aware of the interconnections between health behaviours for a better understanding of how these behaviours affect cognitive health. Governments, particularly in England, could pay more attention to the adverse effects of health behaviours on cognitive health in the ageing population.

**Supplementary Information:**

The online version contains supplementary material available at 10.1186/s12889-022-13785-7.

## Background

Given the absence of curative treatment for dementia, and its associated considerable socioeconomic burden [1, 2], defining strategies to preserve cognitive function in older age has become a pressing public health issue. The World Health Organization has given a strong recommendation for conducting the physical activity and tobacco cessation interventions to reduce the risk of cognitive decline [3]. The international institutions have also highlighted that engaging in multiple positive healthy behaviours can further reduce the risk of cognitive decline [4, 5].

A popular approach to studying the effects of multiple health behaviours on health outcomes is to create an index by summing the number of healthy or unhealthy behaviours that individuals engage in [6–9]. Although this approach provides insight into the cumulative effect of multiple health behaviours, it assumes that the effect of a certain amount of health behaviour is not related to the type of health behaviour endorsed (health behaviours are to be exchangeable). However, health-related behaviours (HRBs) do not occur in isolation, but rather cluster together [10]. This means that a given combination of HRB is more prevalent than would expect if they were independent. The clustering of HRB has implications for public health interventions. The trends in the health behaviour indicators vary over time and across countries. Better awareness of the clustering of HRB is needed to understand what mechanisms these trends reflect and how they affect health outcomes [11]. Furthermore, inter-related behaviours could be effectively targeted by multidimensional interventions that address multifaceted improvements in lifestyle, instead of via separate interventions that target individual behaviours [12]. Evidence has also shown that interventions that tackle multiple behaviours seem to be more cost-effective than these target individual health behaviour [13]. Epidemiological evidence for the effect of HRB clustering on cognitive decline in older age is still emerging. One study in France quantified the latent clusters of several lifestyle behaviours to derive HRB clustering. The results suggested that participants engaging in multiple unhealthy behaviours – including smoking, alcohol abstinence (due to participants’ health problems caused by heavy drinking previously), low physical activity, and low fruit and vegetable consumption, were more likely to have poor memory and poor executive function in late midlife, compared with those who engaged in multiple healthy behaviours [14]. Clustering analysis, however, is specific to the sample. The generalisability of the positive effect of HRB clustering on cognitive ageing to more recent years of data and/or among other ageing populations has yet to be established.

Moreover, although social engagement is a factor for healthy cognitive ageing, the role of regular engagement in social activities as one additional component of HRB clustering has so far been largely neglected in multiple health behaviour research [6–9, 14]. Social engagement is a well-established determinant of health, particularly in older age [15], which benefits health directly and/or indirectly through promoting positive health behaviours and alleviating stress responses [16]. Although Public Health England has recommended that social engagement should be a key intervention for dementia prevention [17], the extent to which social-engagement-related HRB clustering is associated with cognitive ageing remains inconclusive.

Methodological challenges regarding the investigation of the HRB clustering also exist. Due to differences in the definitions and categories of HRBs, as well as the cut-off values employed to identify high-risk behaviours, a direct comparison of research findings in HRB clustering and its associations with health outcomes across countries is usually inapplicable [14]. However, conducting cross-country comparison in behavioural research is still needed, since ageing research needs to be better coordinated across countries, to discover the most cost-effective approaches to maintain older people’s health and well-being [18].

Researchers are currently harmonising databases of sister longitudinal studies of ageing worldwide [19]. These studies are nationally representative, and they commonly incorporate measures of health behaviours and cognitive measures, providing a unique opportunity to conduct a multinational comparison of HRB-related inequalities in cognitive health, on a scale not having done before. Our previous work has thus identified and compared HRB clustering across countries based on these harmonised databases. Apart from smoking, alcohol consumption and physical activity, we included social engagement as one component of the HRB clustering in our previous work [10]. Building on this previous work, the current study aimed to explore the extent to which memory trajectories would vary by HRB clusters within and between countries. We chose to focus on the USA and England firstly. Both countries had high dementia burdens. The ranges of the age-standardised prevalence per 100,000 individuals for Alzheimer’s disease and other dementias for both sexes in 2016 were 700–800 in the USA and 600–700 in England, while in Canada and other Northern European countries, the prevalence was less than 600 [1]. Both countries are top economies in their continental regions but are experiencing labour force ageing [20]. The findings of our study can be instructive for developing the methodology of comparing the effect of HRB clustering on episodic memory between multiple countries quantitatively, and designing common and regional-specific HRB interventions to prevent cognitive ageing and thereby facilitate healthy ageing cross-nationally. A healthy ageing population will be able to transform ageing challenges into productivity and permit older people to contribute to society by staying in the labour market longer [21].

Specifically, our objectives were to examine the association between previously detected HRB clusters and episodic memory trajectories in each country; and to compare the trajectories of episodic memory over age across the HRB clusters between the two countries by quantifying the HRB-related difference in mean values of episodic memory and the age-related rate of slope change in episodic memory across HRB clusters.

## Methods

### Study sample

Data from the Health and Retirement Study (HRS) in the USA [22] and the English Longitudinal Study of Ageing (ELSA) [23], comprising a combined sample of 26,241 participants aged ≥ 50 years in 2010/2011. Ethical approvals were granted from the University of Michigan Institutional Review Board (for HRS) and the London Multicentre Research Ethics Committee (MREC/01/2/91, for ELSA). Informed consent was obtained from all participants. Our longitudinal analysis included data from waves 10–14 (2010–2018; wave 10 was treated as the baseline wave for the current study) in HRS, and waves 5–9 (2010–2018; wave 5 was treated as the baseline wave for the current study) in ELSA. Both samples came from a complex survey design, with respondent-level weights being defined at each wave. Baseline cross-sectional weights (for both HRS and ELSA) and stratification and cluster variables (for HRS only; unavailable in ELSA after waves 1 and 2 of data collection) were used to adjust for bias due to sampling design when conducting analyses. We excluded booster samples who were age-ineligible respondents and had zero values of cross-sectional weight at the baseline wave for the current study (i.e., 2010). Ultimately, 7,354 men and 10,396 women in the USA, as well as 3,769 men and 4,722 women in England, were included for analysis.

### HRB clustering

HRB clustering performed on smoking, alcohol consumption, physical activity and social activity was identified gender-specifically in each country using latent class analysis [24] in our previous study [10]. HRB clusters were identified as follows:

- Multi-HRB cluster: characterised by multiple positive behaviours: ex-/never smoking, moderate drinking, being socially and physically active;
- Inactive cluster: distinguished by infrequent involvement in social and physical activities without other risk behaviours;
- (Ex-)smoking cluster: with current smoking in men and with ex-smoking in women, coupled with excessive drinking, and being socially or physically inactive.

Three clusters including the multi-HRB, inactive and ex-smoking clusters were found in both US and English women; and three clusters including the multi-HRB, inactive and smoking clusters were found in US men. However, only two HRB clusters were found in English men (i.e., multi-HRB and inactive clusters) [10]. All these gender- and country-specific clusters were used in our current study.

### Episodic memory test

Episodic memory was used as a marker of cognitive functioning. Scores from the multiple waves were used. Episodic memory was assessed in a standardised way in each cohort via two-word recall tests: respondents were read a series of 10 words and then asked to immediately recall as many words as possible in any order (immediate recall: range 0–10). After approximately five minutes, respondents were asked to recall as many of the original words as possible in any order (delayed recall: range 0–10) [25]. From these, we summed the number of words recalled (range 0–20), with higher scores indicating better episodic memory.

### Confounders

Baseline variables including birth cohort (year of birth), marital status, educational attainment, household wealth, labour force status, and the presence of any self-reported long-term conditions were considered for adjustment as potential confounders of the HRB clustering and episodic memory associations. The long-term conditions included high blood pressure, diabetes, cancer, lung disease, stroke, heart problems, psychological problems and arthritis.

### Analytic strategy

Baseline missing data in covariates and memory outcomes (see Supplementary Table S1) were excluded but missing data in other waves of data collection during follow-up were not excluded. We analysed our dataset in long format to use all available information in later waves after baseline. Analyses were conducted for men and women separately, given previous findings of substantial gender differences in HRB clustering [10]. The HRB cluster membership uncertainty was maintained by controlling for logged ratios of the average posterior class membership probabilities, as suggested by the three-step method. This method allows the initial mixture model and secondary analyses to be conducted independently, but still maintains the uncertainty in subgroup membership throughout [26].

Means or proportions of baseline HRB clustering, episodic memory scores and confounders, as well as gender- and country-stratified simple relationships between baseline HRB clustering and episodic memory scores, and between each confounder and episodic memory scores were examined, accounting for baseline survey weighting, cluster (not for ELSA) and stratification (not for ELSA). See Supplementary Methods for more details.

To achieve our research aims, main analyses were undertaken within and between countries. Baseline respondent-level weights was also considered. See Supplementary Methods for detailed syntax.

### Within country associations

The longitudinal association between HRB clustering and episodic memory within each country was examined using an age-based multilevel growth curve model (controlling for birth cohort), allowing for random intercepts and slopes for each participant [27]. Age was centred on each sample’s baseline mean to aid interpretation. Age is the metric of time. We controlled for the cohort effect using the year of birth (birth cohort) to build the Age-Cohort model (repeat age model controlling for birth cohort). The interaction between age and birth cohort was statistically non-significant, and thus was not included in the modelling. Both age and age^2^ were included. Each model with a quadratic trend over age was additionally adjusted for marital status, education, wealth, labour force status, and presence of any long-term conditions. The coefficients for the variables of HRB clusters (multi-HRB as reference) indicate relationships between HRB clusters and the level of episodic memory at the centred baseline age. We also allowed for an interaction between age and HRB clustering. A statistically significant age × HRB cluster term suggests that the age-related slope change in episodic memory over the follow-up period varied across HRB clusters. Further, we tested the interaction between age^2^ and HRB clustering, as well as the covariance between intercept and slope.

The following is an example of a multilevel model with a quadratic trend over age, and the interaction between age and HRB cluster only. Memory_ij_ indicates the score of episodic memory in wave i for individual j. HRB_j_ is the time-invariant HRB cluster for individual j. Age_ij_ and age_ij_^2^ are time-varying. Every individual’s memory trajectory is modelled as a function of age, age^2^, HRB cluster, as well as the interaction between age and HRB cluster. The intercept $\beta$_0j_ is made up of two parts: the fixed part ${\gamma }_{00}$, representing the mean intercept; and the random part ${U}_{0j}$, representing individual deviations from the mean intercept. The coefficient for age, ${\beta }_{1j}$, is also made up of two parts: the fixed part ${\gamma }_{10}$, representing the mean slope; the random part ${U}_{1j}$, representing individual deviations from the mean slope. The time-specific residual term or random error for each individual, ${\varepsilon }_{ij}$, is assumed to be normally distributed with a mean at zero and constant over all ages. The random coefficients ${U}_{0j}$ are not estimated directly; instead, the variance of ${U}_{0j}$ captures individual variations in baseline memory. The coefficient ${\beta }_{3-1}$ and ${\beta }_{3-2}$ are the fixed effects of the HRB cluster at baseline. The coefficient ${\beta }_{4}$ is the fixed effect of the interaction between HRB cluster and age and signifies whether ageing trajectories depend on an individual’s HRB cluster.

$${Memory}_{ij}= {\beta }_{0j}+{\beta }_{1j}{age}_{ij}+{{\beta }_{2j}age}_{ij}^{2}+{\beta }_{3-1}{HRB}_{j}+{{\beta }_{3-2}{HRB}_{j}+{\beta }_{4}HRB}_{j}*{age}_{ij}+{\varepsilon }_{ij}$$

$${\beta }_{0j}={\gamma }_{00}+{U}_{0j}$$

$${\beta }_{1j}={\gamma }_{10}+ {U}_{1j}$$

### Between country comparisons

We quantified between-country inequalities in the trajectories of episodic memory by HRB clusters. This required testing whether the effect of HRB clustering on episodic memory significantly differed between countries. This hypothesis was formally tested by including a three-way interaction term (age × HRB cluster × country).

Given the variations in the number of HRB clusters identified in each country [10], only data for participants belonging to the common HRB cluster between countries were combined for analysis. Two separate analyses were performed: (1) a comparison of the multi-HRB (reference) and inactive clusters between English and US men; and (2) a comparison of the multi-HRB (reference), inactive, and ex-smoking clusters between English and US women. A significant three-way interaction term would indicate that the differences in the age-related memory trajectories by HRB cluster (e.g., a protective effect for the multi-HRB cluster versus the other clusters) are not uniform but rather vary between countries. Episodic memory trajectories by HRB cluster for each country from the relevant growth curve estimates were drawn separately to aid interpretation.

All analyses were performed using Stata SE V15.0 [28], with a *P*-value threshold of < 0.05 for statistical significance.

## Results

Table 1 describes the baseline sample characteristics by country and gender. US men had a lower mean score of episodic memory than their English counterparts. Among both men and women, marriage was more common in England than in the USA. US men and women tended to be more highly educated than their English counterparts. The presence of any long-term conditions was prevalent in both countries. The proportion of respondents belonging to the HRB clusters identified varied between countries. The multi-HRB cluster contained the majority of the US (56.3%) and English (77.7%) men. Around 42% of US women were in the ex-smoking cluster, while around 43% of English women were categorised in the multi-HRB cluster. Over 50% of English participants were retired while 48% of US men were still in work. Besides, all covariates were significantly associated with episodic memory at baseline in men and women (Supplementary Table S2).

**Table 1:** Baseline sample characteristics by gender in the USA and England

Variables	USA – Men (*N* = 7354)	USA – Women (*N* = 10,396)	England – Men (*N* = 3769)	England – Women (*N* = 4722)
**Mean (S.E.)**				
**Episodic memory score**	9.6 (0.05)	10.4 (0.04)	10.0 (0.1)	10.4 (0.1)
**Age**	64 (0.1)	65 (0.1)	66 (0.2)	67 (0.2)
**%**				
**HRB clustering**				
Multi-HRB cluster	56.3	31.0	77.7	43.4
Inactive cluster	21.3	26.6	22.3	33.0
Smoking cluster	22.4	-	-	-
Ex-smoking cluster	-	42.3	-	23.7
**Birth cohort**				
Born in 1950–1959	39.1	37.2	31.7	29.3
Born in 1940–1949	33.7	32.2	35.9	33.7
Born in 1930–1939	18.2	18.8	22.6	22.9
Born in 1929 and earlier	8.9	11.9	9.8	14.0
**Marital status**				
Married or partnered	73.6	56.5	77.0	61.0
Separated, divorced or single	20.5	22.8	14.7	16.7
Widowed	5.9	20.7	8.3	22.3
**Education**				
First stage of tertiary or more	37.8	30.0	18.5	10.2
Upper secondary education	48.8	55.5	23.2	17.3
Lower secondary education	9.2	10.7	22.4	22.7
Primary education or less	4.3	3.9	35.9	49.8
**Wealth**				
Highest	26.8	23.2	20.5	16.6
2^nd^	22.6	21.9	19.7	18.7
3^rd^	19.8	19.6	20.2	19.4
4^th^	17.2	19.0	19.1	20.8
Lowest	13.7	16.2	20.5	24.5
**Labour force status**				
Work full-time or part-time	48.0	39.6	37.5	26.4
Unemployed	4.4	3.3	2.0	0.5
Retired	44.1	47.0	53.4	58.4
Disabled	1.9	2.4	5.6	4.7
Not in the labour force	1.6	7.7	1.5	10.0
**Presence of any long-term condition**				
No	10.6	10.3	28.4	23.6
Yes	89.4	89.7	71.6	76.4

*S.E.* Standard error

### Within country associations

Table 2 shows results for the longitudinal associations between HRB clustering and episodic memory trajectories among men (upper panel) and women (lower panel) in each country. For the fixed effects, with regards to intercept, men in the multi-HRB cluster had higher scores of episodic memory than their counterparts in the inactive (USA and England) or smoking (USA) clusters. Over the follow-up period, the declining rate of episodic memory for men in the inactive (England) and smoking (USA) clusters did not systematically differ in the multi-HRB cluster (shown by the non-significant interaction between age and inactive cluster [England] or between age and smoking cluster [USA]). However, among US men, a small but statistically significant difference in the age-related decline was found for participants in the inactive cluster, who showed a lower rate of decline in episodic memory than their counterparts in the multi-HRB cluster (age × inactive cluster: 0.01 [0.001, 0.03]). The interaction between age^2^ and HRB clustering was non-significant and was therefore not included in the current model. In terms of the random effects, coefficients for the variance of episodic memory at the occasional level were at 2.23^2^ (4.97) and 2.26^2^ (5.10), with 58.2% and 52.2% of the unexplained variance in the fully adjusted models by HRB clustering attributable to unobserved individual factors in the USA and England, respectively. The covariance between intercept and slope was non-significant and was therefore not included in the current model.

**Table 2:** Results of fully adjusted multilevel models for associations between HRB clustering and episodic memory trajectories by gender in the USA and England^a^

**Men**	**USA (*N* = 7354)**		**England (*N* = 3769)**	
**Fixed effects**	**b (95%CI)**	***P*-value**	**b (95%CI)**	***P*-value**
**Intercept**	11.79 (11.55, 12.02)	< 0.001	12.07 (11.73, 12.41)	< 0.001
**Age**	-0.07 (-0.08, -0.06)	< 0.001	-0.15 (-0.17, -0.14)	< 0.001
**Age**^**2**^	-0.0033 (-0.0037, -0.0028)	< 0.001	-0.006 (-0.007, -0.004)	< 0.001
**HRB clusters**				
Multi-HRB cluster	Reference		Reference	
Inactive cluster	-0.18 (-0.33, -0.04)	0.010	-0.87 (-1.15, -0.59)	< 0.001
Smoking cluster	-0.33 (-0.47, -0.20)	< 0.001	-	-
**Interactions: HRB clustering x age**				
Inactive cluster	0.01 (0.001, 0.03)	0.029	-0.003 (-0.03, 0.02)	0.789
Smoking cluster	-0.005 (-0.02, 0.01)	0.372	-	
**Random effects**	**S.D. (95%CIs)**		**S.D. (95%CIs)**	
**Level 1: residual**	2.23 (2.21, 2.25)		2.26 (2.21, 2.31)	
**Level 2: intercept**	1.89 (1.84, 1.95)		2.14 (2.05, 2.23)	
**Level 2: age**	0.02 (0.003, 0.11)		0.08 (0.06, 0.10)	
**Women**	**USA (*N* = 10,396)**		**England (*N* = 4722)**	
**Fixed effects**	**b (95%CIs)**	***P*-values**	**b (95%CIs)**	***P*-values**
**Intercept**	12.57 (12.34, 12.80)	< 0.001	13.15 (12.73, 13.57)	< 0.001
**Age**	-0.11 (-0.12, -0.10)	< 0.001	-0.14 (-0.16, -0.13)	< 0.001
**Age**^**2**^	-0.0042 (-0.0046, -0.0038)	< 0.001	-0.006 (-0.007, -0.005)	< 0.001
**HRB clustering**				
Multi-HRB cluster	Reference		Reference	
Inactive cluster	-0.12 (-0.24, -0.001)	0.048	0.07 (-0.15, 0.29)	0.523
Ex-smoking cluster	0.42 (0.31, 0.53)	< 0.001	-1.36 (-1.67, -1.06)	< 0.001
**Interactions: HRB clustering x age**				
Inactive cluster	-0.003 (-0.01, 0.01)	0.564	0.003 (-0.02, 0.02)	0.776
Ex-smoking cluster	0.003 (-0.007, 0.01)	0.543	-0.03 (-0.05, 0.001)	0.058
**Random effects**	**S.D. (95%CIs)**		**S.D. (95%CIs)**	
**Level 1: residual**	2.35 (2.33, 2.37)		2.32 (2.27, 2.38)	
**Level 2: intercept**	1.90 (1.85, 1.95)		2.26 (2.16, 2.36)	
**Level 2: age**	0.05 (0.04, 0.07)		0.05 (0.02, 0.10)	

^a^Each model with a quadratic trend over age was adjusted for birth cohort, marital status, education, wealth, long-term conditions and the interaction between age and HRB clustering.

*95%CI* 95% Confidence interval

For the fixed effects, among US and English women, differences across the HRB clusters were found for the intercept, but not slope, in episodic memory. With regards to intercept, English women within the multi-HRB cluster had higher episodic memory scores than their counterparts within the ex-smoking cluster. US women within the multi-HRB cluster had higher episodic memory scores than their counterparts within the inactive cluster, whereas had lower episodic memory scores than their counterparts within the ex-smoking cluster. The interaction between age^2^ and HRB clustering was non-significant and was therefore not included in the current model. In terms of the random effects, coefficients for the variance of episodic memory at the occasional level were at 2.35^2^ (5.52) and 2.32^2^ (5.38), with 60.5% and 52.2% of the unexplained variance in the fully adjusted models by HRB clustering attributable to unobserved individual factors in the USA and England, respectively. The covariance between intercept and slope was non-significant and was therefore not included in the current model.

### Between country comparisons

Between-country comparisons for the effect of HRB clustering on episodic memory are shown in Tables 3 and 4. Our analyses produced three main findings.

**Table 3:** Results of fully adjusted multilevel models for comparing differences in episodic memory trajectories by HRB clustering and country among men^a^

**Fixed effects**	**England (*N* = 3769) versus USA (*N* = 5730)**	
	**b (95%CI)**	***P*-value**
**Intercept**	11.41 (11.20, 11.63)	< 0.001
**Age**	-0.09 (-0.10, -0.08)	< 0.001
**Age**^**2**^	-0.0041 (-0.0045, -0.0036)	< 0.001
**HRB clustering**		
Multi-HRB	Reference	
Inactive cluster	-0.27 (-0.42, -0.11)	0.001
**Interactions: HRB clustering x age**		
Inactive cluster	0.01 (-0.001, 0.03)	0.066
**Country**		
England versus the USA: multi-HRB	1.29 (1.11, 1.48)	< 0.001
**Interaction: HRB clustering x country**		
England versus the USA: (inactive cluster vs multi-HRB cluster)	-0.56 (-0.85, -0.27)	< 0.001
**Interaction: HRB clustering x country x age**		
England versus the USA: multi-HRB cluster	-0.05 (-0.06, -0.03)	< 0.001
England versus the USA: inactive cluster	-0.07 (-0.09, -0.05)	< 0.001
**Random effects**	**S.D. (95%CIs)**	
**Level 1: residual**	2.26 (2.24, 2.28)	
**Level 2: intercept**	2.03 (1.99, 2.08)	
**Level 2: age**	0.05 (0.03, 0.06)	

^a^ The model with a quadratic trend over age was adjusted for birth cohort, marital status, education, wealth, long-term conditions, country, and interactions between age and HRB clustering, age and country, as well as among age, HRB clustering and country.

*95%CI* 95% Confidence interval

**Table 4:** Results of fully adjusted multilevel models for comparing differences in episodic memory trajectories by HRB clustering and country among women^a^

**Fixed effects**	**England (*N* = 4722) vs USA (N = 10,396)**	
	**b (95%CI)**	***P*-value**
**Intercept**	12.21 (12.01, 12.41)	< 0.001
**Age**	-0.09 (-0.10, -0.18)	< 0.001
**Age**^**2**^	-0.0047 (-0.0050, -0.003)	< 0.001
**HRB clustering**		
Multi-HRB	Reference	
Inactive cluster	-0.17 (-0.30, -0.05)	0.007
Ex-smoking cluster	0.48 (0.37, 0.60)	< 0.001
**Interaction: HRB clustering x age**		
Inactive cluster	-0.003 (-0.01, 0.01)	0.626
Ex-smoking cluster	0.003 (-0.007, 0.01)	0.514
**Country**		
England versus the USA: multi-HRB cluster	1.97 (1.77, 2.16)	< 0.001
**Interactions: HRB clustering x country**		
England versus the USA: (inactive cluster versus multi-HRB cluster)	0.17 (-0.04, 0.37)	0.105
England versus the USA: (ex-smoking cluster versus multi-HRB cluster)	-1.73 (-1.97, -1.49)	< 0.001
**Interactions: HRB clustering x country x age**		
England versus the USA: multi-HRB cluster	-0.04 (-0.05, -0.02)	< 0.001
England versus the USA: inactive cluster	-0.03 (-0.04, -0.01)	0.001
England versus the USA: ex-smoking cluster	-0.06 (-0.07, -0.04)	< 0.001
**Random effects**	**S.D. (95%CIs)**	
**Level 1: residual**	2.35 (2.34, 2.37)	
**Level 2: intercept**	2.06 (2.02, 2.10)	
**Level 2: age**	0.05 (0.04, 0.06)	

^a^ Each model with a quadratic trend over age was adjusted for birth cohort, marital status, education, wealth, long-term conditions, country, and interactions between age and HRB clustering, age and country, as well as among age, HRB clustering and country.

*95%CI* 95% Confidence interval

Firstly, intercepts of episodic memory varied by country within the same HRB cluster. Within the multi-HRB cluster, English men had a higher level of episodic memory than those in the USA (b = 1.29, 95% CI: 1.11, 1.48). A similar finding was found for women (b = 1.97, 95% CI: 1.77, 2.16).

Secondly, differences in the intercept of episodic memory across HRB clusters varied by country. When comparing the difference in mean values of episodic memory between inactive and multi-HRB cluster members, this difference in England was larger for men (b = -0.56, 95% CI: -0.85, -0.27) compared with their US counterparts. Among women, the difference in mean values of episodic memory between ex-smoking and multi-HRB cluster members was larger in England than in the USA (b = -1.73, 95% CI -1.97, -1.49).

Thirdly, differences in the age-related rate of slope change in episodic memory across HRB clusters also varied by country (Supplementary Figure S1). All these differences in declining rates were statistically significant due to negatively significant three-way interactions (age × HRB cluster × country, see Tables 3 and 4). These significant interactions suggested that with increased age, for men and women within the same HRB cluster, the decline in episodic memory was faster for English participants than their US counterparts.

## Discussion

Using longitudinal data from the two ageing cohorts, we examined differences in episodic memory trajectories by three largely consistent HRB clusters. Participants within negative HRB clusters were related to lower episodic memory scores compared with those within the multi-HRB cluster in both the USA and England. However, the effect of HRB clustering on episodic memory varied between countries: episodic memory trajectories declined faster after age 50 in England than in the USA; and the HRB-related difference in mean values of episodic memory was greater in England than in the USA, when comparing memory scores for men within the inactive cluster with those within the multi-HRB cluster, and women within the ex-smoking cluster with those within the multi-HRB cluster.

Our finding of the beneficial effects of multiple health behaviours on cognitive ageing in the USA and England was consistent with previous work, which examined the effects of either HRB clustering or summed HRB indices on cognitive ageing globally [6–9, 14]. For example, one study of 196,383 British older adults found that engaging in three or four positive HRB, namely, not smoking, regular physical activity, healthy diet and moderate alcohol consumption was associated with low risk of incident all-cause dementia regardless of genetic risk profile [9]. Our findings further showed that participants within the inactive cluster, despite having no other risk behaviours other than being physically inactive and not socially engaged (i.e., US and English men, and US women), still had lower episodic memory than those within the multi-HRB cluster. Therefore, even though previous evidence overlooked social engagement as one essential component of multiple healthy behaviours, our study, which involves social activity as one component of HRB clustering, contributes to the literature by demonstrating the importance of engaging in social and physical activities to preserve episodic memory in old age.

We also found variations in the HRB related inequalities in episodic memory between countries: US women within the ex-smoking cluster had better episodic memory function than those within the multi-HRB cluster; whereas this association is in the opposite direction to that identified among English women. This variation might be partially driven by other multiple HRB within the two ex-smoking clusters. In our sample, the English, but not the US, female ex-smokers also had a high probability of being heavy drinkers (consuming > 2 drinks/day) [10]. A UK study showed that women who were ex-smokers and heavy drinkers had a faster cognitive decline in later life than those who were non-smokers and moderate drinkers [29]. Moreover, the effect of smoking cessation on cognitive decline in older age remains unclear [30, 31]. A UK study found that recent ex-smokers still exhibited greater cognitive decline compared to non-smokers, but longer-term ex-smokers (≥ 10 years) showed no difference [32]. Future research could investigate the effect of smoking cessation duration on cognitive function based on the data available in the HRS.

Our between-country comparison showed that although English men and women within the multi-HRB cluster both had better episodic memory than their US counterparts, the age-related decline in episodic memory tended to be faster among English men and women within each HRB cluster than that among their US counterparts. Moreover, the HRB-related difference in mean values of episodic memory was greater in England than the USA, when comparing memory scores for men within the inactive cluster with those within the multi-HRB cluster, and women within the ex-smoking cluster with those within the multi-HRB cluster. It seemed that on average, with increased age after 50 years old, US participants maintained better episodic memory than English participants. A previous study based on the HRS and ELSA data also found that US participants aged ≥ 65 years were cognitively healthier on average than English participants; and that higher levels of education and wealth, lower levels of depressive symptoms, and more aggressive treatment of cardiovascular risks in the USA could be contributing factors [33].

### Limitations

Our findings must be interpreted within the context of the limitations. Firstly, the same variables between countries must be used for data harmonisation, resulting in the exclusion from this study of other country-specific covariates. For example, we were unable to adjust for occupation due to a lack of harmonised information, even though previous evidence shows a significant association between occupation and health behaviours [34], and between occupation and cognitive functioning [35].

Secondly, we only identified HRB clusters at baseline wave (2010) in HRS and ELSA. The HRB clusters might change within individuals over the six-year follow-up period. However, using HRB clusters at baseline only has ensured a clear temporality as those HRB clusters precede the outcome of episodic memory. The risk of opposite causation – memory impairment at baseline leading to behavioural changes, would be reduced.

Thirdly, we conducted complete case analyses by excluding missing data. Participants who dropped out of the study after the baseline wave were more likely to have severe illness than those who remained. The application of the multilevel modelling could handle attrition, wave nonresponse, and unequal time spacing. Although statistical strategies can to some extent address the potential bias caused by missingness, they are not perfect and our findings might still underestimate the association between HRB clustering and episodic memory trajectories.

Fourthly, due to unavailability of the stratification and cluster variables after waves 1 and 2 of data collection in ELSA, simple analyses did not adjust for stratification and cluster. Furthermore, our main analyses in both ELSA and HRS only considered survey weights since the STATA commands for growth curve modelling (*xtmixed*) and survey adjustment (*svy*) cannot be used simultaneously. Therefore, the standard errors produced by these analyses without adjustment for stratification and cluster would probably be smaller than they should be in ELSA and HRS.

Finally, there might be differences in variances across HRB clusters. But we assumed equal variances across HRB clusters in our analyses. Testing this assumption might not be applicable since the HRB clusters are latent groups.

### Implications

Our findings reinforce the suggestion of involving multiple components of HRB in primary prevention of cognitive impairment, as well as in policy recommendations regarding lifestyle and well-being in later life [14]. As different clusters have been identified, older people within different clusters may benefit from different interventions depending on which unhealthy behaviours they partake in. Through including social activity as one component of HRB clustering, which has never done by previous studies, our study highlights that maintaining or increasing engagement in social activities, and so prevent social isolation among the ageing population, should be noted by policymakers and healthcare providers. Findings of the slower decline in episodic memory trajectories, as well as the smaller HRB-related inequality since 2010 among US participants than that among English participants, might be a positive sign for the efforts that the US governments and health care providers made to prevent cognitive impairments at the population level, especially after 2011, when the National Alzheimer’s Project Act had been signed into law in the USA [36]. However, our findings of significant associations between HRB clusters and episodic memory in both countries still emphasise the necessity of facilitating appropriate multiple behavioural change interventions for cognitive preservation at the population level.

## Conclusions

In conclusion, HRB clustering was associated with trajectories of episodic memory in both the USA and England. The effect of HRB clustering on episodic memory seemed larger in England than the USA. Our study highlighted the importance of being aware of the interconnections between health behaviours for a better understanding of how these behaviours affect cognitive health. Governments, particularly in England, could pay more attention to the adverse effects of health behaviours on cognitive health in the ageing population.

## Supplementary Information

**Additional file 1:** **Table S1.** Percentages of missingness in each variable across waves by gender in the USA and England. **Table S2.** Baseline simple relationships between covariates and episodic memory scores among men and women in the USA and England. **Figure S1**. Comparing episodic memory trajectories by gender and country. **Supplementary Methods.** Examples for baseline sample characteristics estimation, baseline simple regression analyses, and main longitudinal analyses in HRS and ELSA.

## Data Availability

The datasets used and/or analysed during the current study are available from the corresponding author on reasonable request.

## Abbreviations

HRB: Health-related behaviour
USA: United States of America
WHO: World Health Organization
HRS: Health and Retirement Study
ELSA: English Longitudinal Study of Ageing
