# Supplementary material for: Comparing relationships between health-related behaviour clustering and episodic memory trajectories in the United States of America and England: a longitudinal study
Source: BMC Public Health. 2022 Jul 16;22:1367. doi: 10.1186/s12889-022-13785-7 (PMC9288697; doi:10.1186/s12889-022-13785-7)
Supplement: Supplementary file 1 — Additional file 1: Table S1. Percentages of missingness in each variable across waves by gender in the USA and England. Table S2. Baseline simple relationships between covariates and episodic memory scores among men and women in the USA and England. Figure S1. Comparing episodic memory trajectories by gender and country. Supplementary Methods. Examples for baseline sample characteristics estimation, baseline simple regression analyses, and main longitudinal analyses in HRS and ELSA. [file 12889_2022_13785_MOESM1_ESM.docx]

**Supplementary documents**

***Table S1 Percentages of missingness in each variable across waves by gender in the USA and England***

| USA – Men | Wave 10 (N=8,777) | Wave 11 (N=7,828) | Wave 12 (N=6,996) | Wave 13 (N=6,068) | Wave 14 (N=4,652) |
| --- | --- | --- | --- | --- | --- |
|  | **%** | **%** | **%** | **%** | **%** |
| Episodic memory score | 2.2 | 2.0 | 3.6 | 4.8 | 12.5 |
| Age | 0.0 | 0.0 | 0.0 | 0.0 | 0.0 |
| HRB clustering | 0.0 |  |  |  |  |
| Age | 0.0 |  |  |  |  |
| Birth cohort | 0.0 |  |  |  |  |
| Marital status | 0.0 |  |  |  |  |
| Education | 0.0 |  |  |  |  |
| Wealth | 0.0 |  |  |  |  |
| Labour force status | 0.0 |  |  |  |  |
| Long-term conditions | 4.2 |  |  |  |  |
| USA – Women | **Wave 10 (N=11,560)** | **Wave 11 (N=10,566)** | **Wave 12 (N=9,668)** | **Wave 13 (N=8,576)** | **Wave 14 (N=6,705)** |
|  | **%** | **%** | **%** | **%** | **%** |
| Episodic memory score | 2.0 | 1.6 | 3.3 | 4.2 | 10.5 |
| Age | 0.0 | 0.0 | 0.0 | 0.0 | 0.0 |
| HRB clustering | 0.0 |  |  |  |  |
| Age | 0.0 |  |  |  |  |
| Birth cohort | 0.0 |  |  |  |  |
| Marital status | 0.0 |  |  |  |  |
| Education | 0.0 |  |  |  |  |
| Wealth | 0.0 |  |  |  |  |
| Labour force status | 0.0 |  |  |  |  |
| Long-term conditions | 3.8 |  |  |  |  |
| England – Men | **Wave 5 (N=4,010)** | **Wave 6 (N=3,545)** | **Wave 7 (N=3,083)** | **Wave 8**  **(N=2,721)** | **Wave 9**  **(N=2,377)** |
|  | **%** | **%** | **%** | **%** | **%** |
| Episodic memory score | 1.8 | 2.1 | 2.8 | 0.0 | 3.9 |
| Age | 0.0 | 0.0 | 0.0 | 0.0 | 0.0 |
| HRB clustering | 0.0 |  |  |  |  |
| Age | 0.0 |  |  |  |  |
| Birth cohort | 0.0 |  |  |  |  |
| Marital status | 0.0 |  |  |  |  |
| Education | 0.2 |  |  |  |  |
| Wealth | 0.7 |  |  |  |  |
| Labour force status | 0.0 |  |  |  |  |
| Long-term conditions | 0.0 |  |  |  |  |
| England – Women | **Wave 5 (N=4,973)** | **Wave 6 (N=4,415)** | **Wave 7 (N=3,892)** | **Wave 8**  **(N=3,441)** | **Wave 9**  **(N=3,083)** |
|  | **%** | **%** | **%** | **%** | **%** |
| Episodic memory score | 1.8 | 1.7 | 2.5 | 0.0 | 3.6 |
| Age | 0.0 | 0.0 | 0.0 | 0.0 | 0.0 |
| HRB clustering | 0.0 |  |  |  |  |
| Age | 0.0 |  |  |  |  |
| Birth cohort | 0.0 |  |  |  |  |
| Marital status | 0.0 |  |  |  |  |
| Education | 0.3 |  |  |  |  |
| Wealth | 0.9 |  |  |  |  |
| Labour force status | 0.0 |  |  |  |  |
| Long-term conditions | 0.0 |  |  |  |  |

***Table S2 Baseline simple relationships between covariates and episodic memory scores among men and women in the USA and England***

| Variables | USA – Men  (N=7354) | USA – Women (N=10396) | England – Men (N=3769) | England – Women (N=4722) |
| --- | --- | --- | --- | --- |
|  | b (95%CIs) | b (95%CIs) | b (95%CIs) | b (95%CIs) |
| HRB clustering |  |  |  |  |
| Multi-HRB – Reference |  |  |  |  |
| Inactive cluster | -0.43 (-0.62, -0.24)^*^ | -0.005 (-0.17, 0.16) | -2.11 (-2.38, -1.84)^*^ | 0.13 (-0.10, 0.37) |
| Smoking cluster | -0.88 (-1.07, -0.70)^*^ |  |  |  |
| Ex-smoking cluster |  | 1.14 (0.99, 1.30)^*^ |  | -3.04 (-3.31, -2.77)^*^ |
| Age (Mean (S.D.)) | -0.097 (-0.104, -0.090)^*^ | -0.10 (-0.11, -0.09)^*^ | -0.16 (-0.17, -0.15)^*^ | -0.18 (-0.19, -0.17)^*^ |
| Birth cohort |  |  |  |  |
| Born in 1950-1959 – Reference |  |  |  |  |
| Born in 1940-1949 | -0.09 (-0.27, 0.10) | 0.05 (-0.11, 0.20) | -0.90 (-1.17, -0.63)^*^ | -0.67 (-0.92, -0.42)^*^ |
| Born in 1930-1939 | -1.36 (-1.54, -1.17)^*^ | -1.27 (-1.43, -1.10)^*^ | -2.57 (-2.86, -2.28)^*^ | -2.52 (-2.80, -2.25)^*^ |
| Born in 1929 and earlier | -3.19 (-3.42, -2.94)^*^ | -3.43 (-3.63, -3.22)^*^ | -4.50 (-4.90, -4.09)^*^ | -5.09 (-5.43, -4.75)^*^ |
| Marital status |  |  |  |  |
| Married or partnered – Reference |  |  |  |  |
| Separated, divorced or single | -0.38 (-0.58, -0.18)^*^ | -0.66 (-0.82, -0.50)^*^ | -0.69 (-1.01, -0.36)^*^ | -0.27 (-0.55, 0.02) |
| Widowed | -1.72 (-2.01, -1.44)^*^ | -1.72 (-1.87, -1.56)^*^ | -2.02 (-2.42, -1.62)^*^ | -2.25 (-2.51, -1.99)^*^ |
| Education |  |  |  |  |
| First stage of tertiary or more – Reference |  |  |  |  |
| Upper secondary education | -1.46 (-1.62, -1.30)^*^ | -1.40 (-1.55, -1.25)^*^ | -1.04 (-1.47, -0.62)^*^ | -0.92 (-1.48, -0.36)^**^ |
| Lower secondary education | -3.15 (-3.39, -2.91)^*^ | -3.19 (-3.40, -2.98)^*^ | -1.82 (-2.16, -1.49)^*^ | -1.54 (-1.95, -1.14)^*^ |
| Primary education or less | -3.88 (-4.19, -3.57)^*^ | -4.01 (-4.29, -3.72)^*^ | -3.27 (-3.62, -2.93)^*^ | -3.88 (-4.26, -3.49)^*^ |
| Wealth |  |  |  |  |
| Highest – Reference |  |  |  |  |
| 2^nd^ | -0.70 (-0.92, -0.48)^*^ | -0.48 (-0.69, -0.28)^*^ | -0.64 (-0.98, -0.30)^*^ | -0.88 (-1.22, -0.53)^*^ |
| 3^rd^ | -1.11 (-1.34, -0.89)^*^ | -1.11 (-1.31, -0.91)^*^ | -1.23 (-1.57, -0.89)^*^ | -1.46 (-1.80, -1.11)^*^ |
| 4^th^ | -1.35 (-1.58, -1.12)^*^ | -1.56 (-1.77, -1.36)^*^ | -1.71 (-2.06, -1.36)^*^ | -2.10 (-2.44, -1.76)^*^ |
| Lowest | -1.83 (-2.07, -1.58)^*^ | -2.15 (-2.36, -1.95)^*^ | -2.34 (-2.70, -1.99)^*^ | -2.71 (-3.05, -2.38)^*^ |
| Labour force status |  |  |  |  |
| Work full-time or part-time – Reference |  |  |  |  |
| Unemployed | -0.94 (-1.31, -0.57)^*^ | -0.80 (-1.20, -0.40)^*^ | -0.80 (-1.73, 0.14) | -0.45 (-1.99, 1.10) |
| Retired | -2.01 (-2.18, -1.83)^*^ | -1.92 (-2.09, -1.74)^*^ | -1.65 (-1.91, -1.39)^*^ | -1.68 (-1.97, -1.40)^*^ |
| Disabled | -2.18 (-2.70, -1.65)^*^ | -2.54 (-3.04, -2.05)^*^ | -2.33 (-2.88, -1.77)^*^ | -2.93 (-3.60, -2.27)^*^ |
| Not in labour force | -0.73 (-1.32, -0.15)^***^ | -1.70 (-2.01, -1.39)^*^ | -1.47 (-2.64, -0.31)^***^ | -1.28 (-1.78, -0.77)^*^ |
| Long-term conditions |  |  |  |  |
| No – Reference |  |  |  |  |
| Yes | -1.05 (-1.32, -0.79)^*^ | -1.45 (-1.69, -1.21)^*^ | -1.08 (-1.33, -0.82)^*^ | -1.31 (-1.57, -1.06)^*^ |

*^*^ P-value<0.001; ^**^ P-value<0.01 ^***^ P-value<0.05*

***Figure S1 Comparing episodic memory trajectories by gender and country***

| ***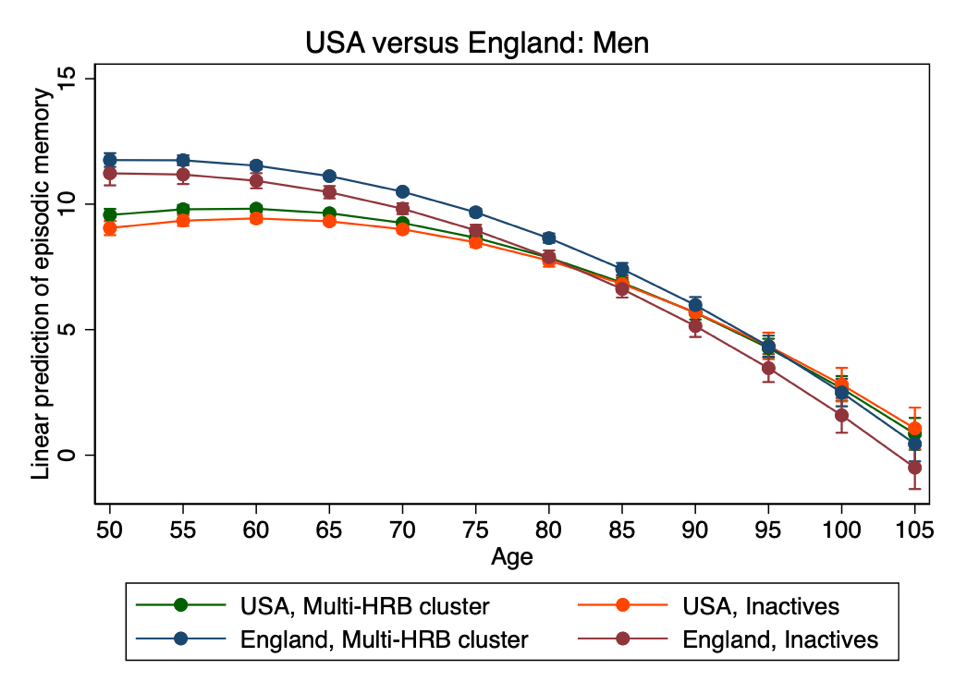*** | ***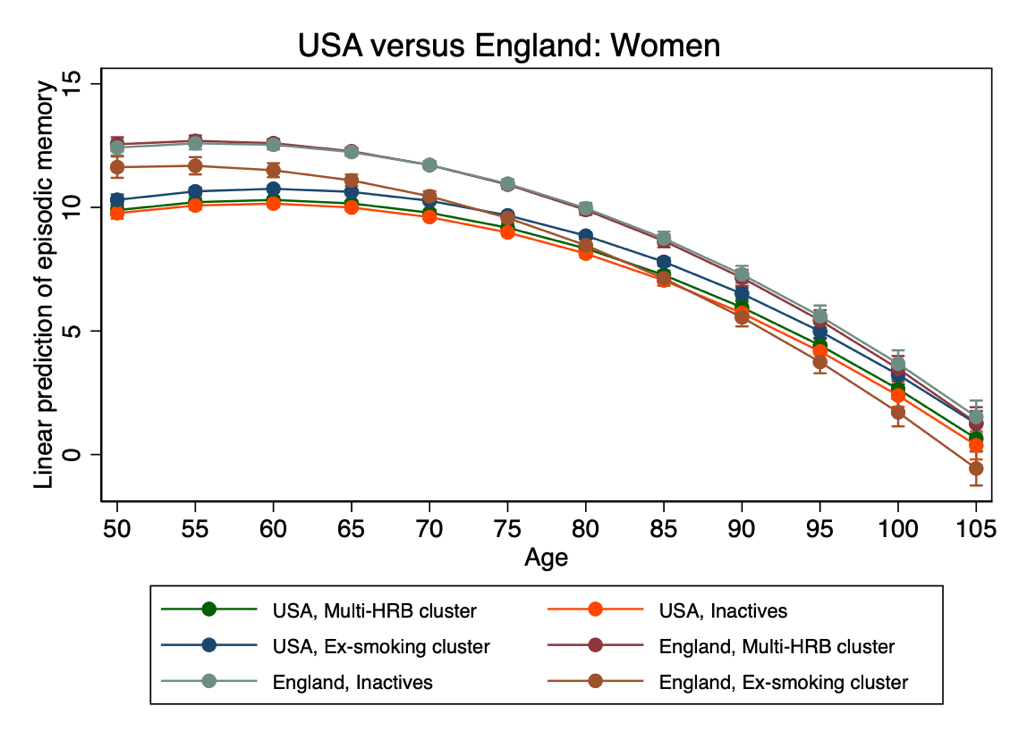*** |
| --- | --- |

**Supplementary Methods – Examples for baseline sample characteristics estimation, baseline simple regression analyses, and main longitudinal analyses in HRS and ELSA**

**For HRS:**

**Baseline sample characteristics estimation and simple regression analyses**

svyset secu [pweight=mwgtr], strata(stratum)

svy: proportion r_education

svy: regress comb10 i.clusterm

**Main longitudinal analyses in men**

wptop=mwgtr*ptop

*[ptop is attained from the three-step approach]*

xtmixed comb c.agecen c.agecen#c.agecen i.coh i.clusterm c.agecen#i.clusterm i.rmstat10 i.r_education i.wealth_10 i.r_lbrf10 i.ncd10 || hhidpn: agecen if miss1number==0, pweight(wptop)

**For ELSA:**

**Baseline sample characteristics estimation and simple regression analyses**

svyset [pweight=r5cwtresp]

svy: proportion r_education

svy: regress comb5 i.clusterm

**Main longitudinal analyses in men**

wptop=r5cwtresp*ptop

*[ptop is attained from the three-step approach]*

xtmixed comb c.agecen c.agecen#c.agecen i.coh i.clusterm c.agecen#i.clusterm i.rmstat5 i.r_education i.wealth_5 i.r_lbrf5 i.ncd5 || idauniq: agecen if miss1number==0, pweight(wptop)
